# Supplementary material for: Lipophilic Substances of the Leaves and Inflorescences of Centaurea scabiosa L.: Their Composition and Activity Against the Main Protease of SARS-CoV-2
Source: Molecules. 2025 Nov 27;30(23):4568. doi: 10.3390/molecules30234568 (PMC12692864; doi:10.3390/molecules30234568)
Supplement: Supplementary file 1 [file molecules-30-04568-s001.zip › molecules-3974117-supplementary.pdf]

Supplementary Materials

Table S1. Content of hydrocarbons in the unsaponifiable residue of lipophilic extracts of *Centaurea scabiosa* inflorescences calculated for the mass of raw material.

Component	RT(min)	Content, mg %		
		**Hexane extract**	**Extract with MTBE after hexane**	**MTBE extract**
Dodecane, n-	10.506	0.73	0.12	0.81
Tetradecane, n-	13.285	1.07	0.39	1.53
Pentadecene	14.662	0.5	Nd*	0.48
Pentadecane	14.756	0.35	0.17	0.48
Hexadecane	15.774	0.42	0.2	0.43
Heptadecane	16.915	0.25	0.22	0.4
Octadecane, n-	17.997	1.07	0.17	1.86
Neophytadien	18.335	0.15	0.02	0.18
Nonadecane	18.932	0.37	0.24	0.56
Eicosane, n-	20.019	0.35	0.09	0.43
Heneicosane, n-	21.145	0.47	0.36	0.54
Docosane, n-	21.867	0.68	0.42	1.07
Tricosane, n-	22.741	10.13	2.26	12.14
Tetracosane, n-	23.564	0.78	1.15	2.85
Pentacosene	24.365	0.38	0.15	0.57
Pentacosane, n-	24.380	13.37	4.59	17.6
13-Methylheptacosane	26.119	0.58	0.25	0.77
Hexacosane, n-	25.138	2.1	1.26	2.98
Heptacosane, n-	25.910	36.24	12.54	42.38
15-Methylnonacosane	26.119	0.71	Nd	0.69
Octacosene	26.580	0.09	Nd	0.06
Octacosane	26.603	5.11	0.56	5.57
Squalene	26.795	2.44	1.84	4.99
2-Methyloctacosane	27.044	0.52	Nd	0.49
Nonacosene	27.137	0.68	0.23	0.76
Nonacosane	27.361	51.23	12.19	62.52
7-Methylnonacosane	27.614	0.48	0.17	0.5
Triacontane	28.069	4.42	1.33	4.54
Hentriacontene	28.699	Nd	Nd	0.3
Hentriacontane	28.733	31.23	4.95	37.52
15- Methylhentriacontane	29.498	2.47	0.38	3.21
Dotriacontane	29.934	7.13	0.77	6.7
Tritriacontane	31.101	1.56	0.51	1.88
Tetratriacontane	31.881	0.82	Nd	0.79
Pentatriacontane	32.249	1.09	Nd	1.32
Heptatriacontane	33.376	0.85	Nd	0.78

Nd – Not detected, RT - retention time, MTBE - methyl tert-butyl ether

0.01 mg% 70.0 mg%

Table S2. Concentrations of hydrocarbons of the unsaponifiable residue of lipophilic extracts of *Centaurea scabiosa* leaves calculated for the mass of raw material.

Component	RT(min)	Content, mg %		
		Hexane extract	Extract with MTBE after hexane	MTBE extract
Dodecane, n-	10.506	0.78	0.11	0.85
Tetradecane, n-	13.285	1.02	0.02	1.06
Pentadecane	14.756	0.47	Nd	0.54
Hexadecane	15.774	2.11	0.32	2.45
Heptadecane	16.915	0.75	0.21	0.9
Octadecane, n-	17.997	1.1	0.14	1.7
Neophytadien	18.335	0.45	0.12	0.58
Nonadecane	18.932	0.57	0.22	0.76
Eicosane, n-	20.019	0.38	0.12	0.53
Heneicosane, n-	21.145	0.29	0.06	0.34
Docosane, n-	21.867	0.58	0.05	0.67
Tricosane, n-	22.741	1.63	0.26	2.14
Tetracosane, n-	23.564	0.88	0.35	1.65
Pentacosene	24.365	0.08	Nd	0.07
Pentacosane, n-	24.380	3.37	0.59	4.6
13-Methylheptacosane	26.119	0.59	Nd	0.56
Hexacosane, n-	25.138	1.18	0.27	1.58
Heptacosane, n-	25.910	16.28	2.58	12.88
15-Methylnonacosane	26.119	0.57	Nd	0.59
Octacosene	26.580	0.07	Nd	0.07
Octacosane	26.603	3.21	0.56	3.64
Squalene	26.795	1.48	1.64	3.46
2-Methyloctacosane	27.044	0.28	Nd	0.29
Nonacosene	27.137	0.48	0.03	0.54
Nonacosane	27.361	31.23	4.95	42.52
7-Methylnonacosane	27.614	0.28	Nd	0.3
Triacontane	28.069	2.47	0.38	2.59
Hentriacontene	28.699	0.31	Nd	0.38
Hentriacontane	28.733	37.3	4.47	42.84
15- Methylhentriacontane	29.498	2.86	0.28	3.26
Dotriacontane	29.934	12.13	1.72	14.3
Tritriacontane	31.101	1.09	0.11	0.94
Tetratriacontane	31.881	0.53	Nd	0.48
Pentatriacontane	32.249	1.05	0.12	1.21

Nd – Not detected, RT - retention time, MTBE - methyl tert-butyl ether

0.01 mg% 50.0 mg%

Table S3. Content of free and bound acid constituents in lipophilic extracts of *Centaurea scabiosa* inflorescences calculated for the mass of raw material (mg%).

Extragent		Hexane		MTBE/Hexane		MTBE	
**Component**	**RT***	**Free**	**Bound**	**Free**	**Bound**	**Free**	**Bound**
Caproic	6.226	1.16	Nd	Nd	0.19	Nd	Nd
4-Methoxybenzoic	13.014	2.32	Nd	1.48	Nd	4.76	Nd
Octanoic	9.539	3.7	3.09	0.32	0.56	3.89	3.06
Decanoic	12.471	1.22	1.07	0.35	0.53	1.57	1.63
8-oxooctanoic	12.593	1.14	Nd	0.23	Nd	1.33	Nd
Hydroxyoctanoic	13.452	1.06	Nd	0.21	Nd	1.31	Nd
8-Oxononanoic	13.727	0.72	Nd	Nd	Nd	0.69	Nd
9-Oxononanoic	13.958	5.93	5.21	0.49	0.2	5.46	5.19
Octandioic	14..095	Nd	Nd	Nd	0.16	Nd	0.2
9-Oxodecanoic	15.034	7.5	1.61	0.26	Nd	1.08	Nd
Dodecanoic	15.070	7.52	12.52	0.99	1.06	5.82	5.3
Nonanedioic	15.358	7.1	0.37	1.44	0.57	7.18	0.86
10-oxo-8-decenoic	15.777	1.52	1.04	0.85	0.04	2.53	1.07
10-oxo-7-decenoic	15.943	4.86	6.5	0.65	0.28	4.72	5.64
10-Oxoundecanoic	16.239	0.96	Nd	0.09	Nd	1.11	Nd
Tridecanoic	16.300	0.22	Nd	0.03	Nd	0.26	Nd
11-oxo-9-undecenoic	17.120	1.21	Nd	Nd	Nd	1.19	Nd
Miristoleic	17.264	Nd	1.9	Nd	0.2	3.6	0.98
Tetradecanoic	17.387	6.53	8.43	1.02	0.76	5.28	4.24
(Z)-10-pentadecenoic	18.304	0.95	0.04	0.03	0.19	1.08	0.24
Pentadecanoic	18.463	3.75	2.62	0.47	0.44	1.93	1.2
9-Hexadecenoic	19.300	5.45	30.06	0.94	2.38	39.37	19.53
Hexadecanoic	19.553	92.84	115.4	24.78	13.28	103.8	79.4
Cyclopropaneoctanoic, 2-hexyl-	20.354	1.65	1.72	Nd	Nd	1.48	1.85
Heptadecanoic	20.470	3.97	0.95	0.53	0.29	1.84	1.04
Linoleic	21.149	5.51	210.86	2.2	14.68	62.23	169.53
Oleic	21.199	16.02	115.27	1.42	7.17	18.33	107.48
Linolenic	21.199	4.14	27.08	0.54	3.1	3.18	40.3
Octadecanoic	21.423	21.87	28.66	4.48	1.99	18.53	18.24
Vaccenic	21.678	Nd	0.44	Nd	Nd	Nd	0.38
Octadecatrienic	22.210	Nd	Nd	0.14	Nd	Nd	Nd
Octadecatrienic	22.289	Nd	Nd	0.28	Nd	0.25	Nd
Octadecatrienic	22.294	Nd	Nd	0.16	Nd	Nd	3.48
Nonadecanoic	22.304	0.82	0.14	Nd	Nd	0.84	0.16
Gadoleic	22.975	Nd	9.31	Nd	0.78	Nd	8.45
Oxiraneoctanoic, 3-octyl-	22.751	1.57	Nd	Nd	Nd	1.49	Nd
Cis-13- eicosenic	23.112	1.97	2.66	0.42	0.47	3.24	2.05
Eicosanoic	23.184	20.58	26.51	2.54	2.06	10.4	11.14
Heneicosanoic	24.000	3.15	1.57	0.42	0.18	1.34	1.82
Octadecane-10,12-dien-9-onic	24.152						
Octadecanedioic	24.231	1.24	2.25	0.44	2.09	2.12	2.12
Docosanoic	24.802	2.19	4.94	0.04	0.03	2.45	3.98
Tricosanoic	25.560	11.99	13.49	2.04	1.39	7.39	8.54
Eicosanedioic	25.791	4.22	2.58	0.62	0.36	2.23	1.26
2-Hydroxidocosanoic	Nd	2.64	1.7	0.4	0.33	Nd	1.65

Tetracosanoic	26.311	Nd	Nd	0.38	Nd	0.37	Nd
Pentacosanoic	27.018	10.8	11.46	1.44	0.75	7.16	5.66
2-Hydroxitricosanoic	Nd	2.78	2.05	0.32	0.17	1.07	Nd
Hexacosanoic	27.762	Nd	Nd	0.32	Nd	Nd	Nd
Heptacosanoic	28.592	3.34	3.66	0.44	0.16	1.63	1.45
Octacosanoic	29.552	1.03	Nd	Nd	Nd	0.98	Nd
Triacontanoic	31.992	3.42	5.26	0.21	Nd	2.01	1.11

RT* - retention time given for methyl esters, Nd – Not detected, MTBE - methyl tert-butyl ether

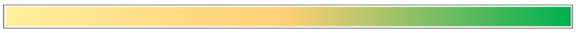

0.01 mg% 220.0 mg%

Table S4. Content of free and bound acid constituents in lipophilic extracts of *Centaurea scabiosa* leaves calculated for the mass of raw material (mg%).

Extragent		Hexane		MTBE/Hexane		MTBE	
**Component**	**RT**	**Free**	**Bound**	**Free**	**Bound**	**Free**	**Bound**
4-Octenoic	9.144	Nd	2.94	Nd	Nd	Nd	1.7
Octanoic	9.539	0.74	Nd	0.8	Nd	1.65	Nd
Decanoic	12.471	1.5	0.87	0.36	0.28	1.89	1.13
4-Oxononanoic	12.869	0.12	Nd	1.88	Nd	2.85	Nd
4-Methoxyacetophenon	12.969	Nd	Nd	3.52	Nd	2.78	Nd
Cinnamic	13.137	Nd	Nd	2.97	Nd	3.08	Nd
4-Methoxybenzoic	13.250	Nd	Nd	8.75	Nd	4.76	Nd
4-Methoxyphenylacetic	13.757	Nd	Nd	5.88	Nd	6.09	Nd
9-Oxononanoic	13.958	Nd	Nd	5.79	4.42	5.76	4.29
Octanedioic	14.095	3.83	1.48	3.67	1.9	3.91	2.13
1,4-Benzendicarboxylic	14.853	Nd	Nd	3.39	1.92	3.43	1.62
9-Oxodecanoic	15.034	1.22	1.43	1.59	1.24	4.39	2.39
Dodecanoic	15.070	7.37	4.27	1.35	1.12	7.69	2.23
Nonanedioic	15.358	14.36	2.21	25.84	6.12	34.88	5.63
3-Oxo-1,8-octanedicarboxylic	15.367	Nd	Nd	Nd	Nd	Nd	3.78
10-oxo-8-decenoic	15.777	Nd	Nd	2.63	Nd	2.13	Nd
p-Methoxycinnamic	15.936	0.15	Nd	3.4	3.34	6.62	2.98
10-oxo-8-decenoic	15.943	Nd	Nd	Nd	3.42	Nd	Nd
Decandioic	16.535	Nd	Nd	1.76	1.21	1.43	1.58
Tetradecenoic	16.932	1.85	Nd	Nd	Nd	1.76	Nd
Tetradecenoic	17.019	4.31	Nd	Nd	Nd	3.98	Nd
Tetradecanoic	17.387	13.11	1.61	8.71	15.08	2.7	8.43
Undecandioic	17.766	Nd	Nd	5.49	Nd	4.58	Nd
9-Hydroxypentadecanoic	17.835	Nd	Nd	1.94	1.29	1.88	1.95
(Z)-10-pentadecenoic	18.304	1.41	Nd	Nd	2.63	1.5	2.46
Pentadecanoic	18.463	5.02	3.48	3.58	5.3	3.33	5.45
Dodecandioic	18.730	Nd	Nd	11.98	Nd	9.89	Nd
14-Methylpentadecanoic	18.850	3.56	Nd	Nd	Nd	4.88	Nd
3,4-Dimethoxy cinnamic	19.127	Nd	Nd	3.4	1.13	4.11	1.8
9-Hexadecenoic	19.300	60.02	6.56	Nd	Nd	58.17	22.21
7-Hexadecenoic	19.445	Nd	Nd	2.64	2.77	2.35	2.84
Hexadecanoic	19.553	159	70.35	179.6	181.48	165.7	109.92
9,10-methylene-hexadecanoic	19.981	Nd	2.28	Nd	Nd	Nd	Nd
Heptadecanoic	20.470	4.14	2.28	4.47	4.03	8.75	4.85
2-Hydroxyhexadecanoic	20.686	Nd	2.41	1.58	0.86	1.39	2.11
Linoleic	21.149	261.1	63.38	30.44	87.26	28.15	130.57
Oleic	21.199	46.04	11.4	11.28	55.4	18.83	26.21
Linolenic	21.199	18.43	3.35	20.68	16.32	34.76	10.04
Octadecanoic	21.423	40.04	9.51	17.05	13.73	13.28	11.3
Vaccenic	21.678	2.07	2.14	Nd	Nd	2.28	2.03
Nonadecanoic	22.304	0.97	Nd	Nd	Nd	1.06	Nd
Pimar-8-en-18-oic	22.406	Nd	2.95	Nd	Nd	Nd	2.02
Pimar-7-en-18-oic	22.486	Nd	2.41	Nd	Nd	Nd	9.71
3-Hydroxystearic	22.787	Nd	Nd	4.91	3.53	3.98	3.64
Eicosadienoic	22.973	Nd	Nd	1.91	3.38	Nd	Nd

9,10-Epoxyoctadecanoic	22.506	1.68	Nd	Nd	Nd	1.59	Nd
Cis-13- eicosenic	23.112	2.29	Nd	Nd	Nd	1.89	Nd
Eicosanoic	23.184	26.05	26.24	25.11	6.47	9.08	3.07
Dehydroabietic	23.237	Nd	12.91	Nd	Nd	Nd	8.96
9cis,11trans,13trans.-octadecatrienoic	23.640	1.76	Nd	7.84	Nd	Nd	Nd
Octadecan-9,10-dien-13-onic	23.930	Nd	2.68	Nd	Nd	Nd	Nd
Heneicosanoic	24.000	2.64	Nd	Nd	Nd	3.22	Nd
Octadecan- 10,12-dien-9-onic	24.152	Nd	2.14	Nd	Nd	Nd	Nd
Octadecanedioic	24.231	Nd	Nd	2.79	Nd	4	Nd
Docosanoic	24.802	18.83	3.08	24.82	7.52	40.23	3.15
Tricosanoic	25.560	4.58	1.61	7.69	3.21	7.75	1.82
15-Tetracosenoic	26.045	1.85	2.68	Nd	Nd	2.02	4.35
Tetracosanoic	26.311	14.78	4.82	24.69	10.08	41.1	3.49
Pentacosanoic	27.018	2.9	2.41	5.1	Nd	7.32	1.9
2-Hydroxytetracosanoic	27.23	0.98	0.56	4.87	1.98	5.04	2.23
Hexacosanoic	27.762	24.22	2.02	9.9	3.54	50.9	4.98
Heptacosanoic	28.592	3.97	Nd	Nd	Nd	4.79	Nd
Octacosanoic	29.552	26.16	9.11	30.61	5.09	22.91	11.28
Nonacosanoic	30.671	2.53	Nd	3.24	Nd	5.3	Nd
Triacontanoic	31.992	20.28	Nd	22.5	2.45	24.26	Nd
Dotriacontanoic	31.992	4.14	2.28	4.47	4.03	8.75	4.85

RT* - retention time given for methyl esters, Nd – Not detected, MTBE - methyl tert-butyl ether

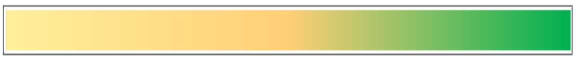

0.01 mg% 270.0 mg%
